# Comparison of cyanoacrylate closure and radiofrequency ablation for the treatment of incompetent great saphenous veins: 36-Month outcomes of the VeClose randomized controlled trial

**DOI:** 10.1177/0268355518810259

**Published:** 2018-11-07

**Authors:** Nick Morrison, Raghu Kolluri, Michael Vasquez, Monte Madsen, Andrew Jones, Kathleen Gibson

**Affiliations:** 1 Center for Vein Restoration, Morrison Vein Institute, Scottsdale, AZ, USA; 2 OhioHealth, Riverside Methodist Hospital, Columbus, USA; 3 Venous Institute of Buffalo, Buffalo, USA; 4 Medtronic Santa Rosa, Santa Rosa, USA; 5 Inovia Vein Specialty Center, Bend, USA; 6 Lake Washington Vascular, Bellevue, USA

**Keywords:** Radiofrequency ablation, endovenous technique, great saphenous vein

## Abstract

**Objective:**

To evaluate the 36-month efficacy and safety of cyanoacrylate closure for the treatment of incompetent great saphenous veins in comparison with radiofrequency ablation.

**Methods:**

In this multicenter, prospective, randomized controlled trial, 222 symptomatic subjects with incompetent great saphenous veins were assigned to either cyanoacrylate closure or radiofrequency ablation. The primary endpoint, complete closure of the target great saphenous vein, was determined using duplex ultrasound examination starting from three-month visit.

**Results:**

At month 36, the great saphenous vein closure rates were 94.4% for the cyanoacrylate closure group and 91.9% for the radiofrequency ablation group. Stable improvement in symptoms and quality of life was observed in both groups. Adverse event rates between the 24- and 36-month visits were similar between the groups as were serious adverse events which were infrequent and judged unrelated to either the device or the procedure in both groups.

**Conclusions:**

This trial continues to demonstrate the safety and efficacy of cyanoacrylate closure for the treatment of great saphenous vein incompetence with great saphenous vein closure rate at 36 months similar to that of radiofrequency ablation, indicating non-inferiority of cyanoacrylate closure to radiofrequency ablation. The improvement in quality of life outcomes were also sustained and similar between the two treatment groups.

## Introduction

Chronic venous disorder (CVD) is a common disease with an estimated worldwide prevalence of 83.6% according to the epidemiologic study by Rabe et al.^1^ CVD symptoms can range from mild discomfort to ulceration and can cause severe disability. CVD also has an effect on patients’ quality of life (QoL)^2^ resulting in considerable health care costs.^3^ The treatment of CVD has undergone a paradigm shift within the last two decades from conventional surgical therapy to minimally invasive endovenous, thermal ablation techniques such as radiofrequency ablation (RFA) and endovenous laser ablation therapy (EVLT).

Although thermal ablation leads to high closure rates and early patient recovery, it does require tumescent anesthesia (TA). The necessary multiple needle sticks can be uncomfortable for the patient with post-procedural ecchymosis and pain commonly reported. Newer non-thermal non-tumescent (NTNT) techniques, including cyanoacrylate closure (CAC), mechanochemical ablation, and proprietary endovenous microfoam, do not require TA or heat energy, and may avoid these associated negative effects.^4,5^

The VenaSeal™ Closure System, an NTNT technique using CAC, received CE mark in September 2011. It was subsequently approved by the US Food and Drug Administration (FDA) in February 2015 through the premarket approval process “for the permanent closure of superficial truncal veins, such as the great saphenous vein (GSV), through endovascular embolization with coaptation”.^6^ Prior to initiating the current VeClose trial, the device was shown to be safe, effective, and feasible for the treatment of saphenous vein incompetence by two prospective clinical studies: a single-center, first-in-human feasibility study with safety and efficacy data reported up to three years^7–9^ and a single-arm, multicenter, European cohort European Sapheon™ Closure System Observational Prospective (eSCOPE) study.^10^ The initial three-month outcomes of the VeClose trial reported non-inferiority of CAC to RFA with GSV closure rates of 99% for the CAC group and 96% for the RFA group.^11^ By month 12, CAC and RFA demonstrated nearly identical, high occlusion rates in the target veins (96.8% in the CAC group and 95.9% in the RFA group), with similar improvements in QoL scores. However, time to complete occlusion was shorter and freedom from recanalization was higher with CAC than with RFA.^5^ By month 24, the closure rates for CAC and RFA were also equivalent (95.3% and 94.0%, respectively) and the rate of freedom from recanalization remained higher in the CAC group, demonstrating continued non-inferiority of CAC to RFA.^12^

In the WAVES study, one-year results demonstrated the safety and efficacy of CAC for the treatment of GSVs up to 20 mm in diameter, small saphenous veins (SSV), and/or accessory saphenous veins, with an occlusion rate of 98% in all veins. ^13^While there is good 12- and 24-month evidence for the use of CAC in incompetent GSV treatment, longer term studies are needed to further establish the safety and efficacy of this treatment modality. In the current 36-month follow-up of the VeClose trial, we report the efficacy and safety of the CAC device in terms of closure rates, safety, symptom scores, and QoL measurements.

## Methods

### Study design and participants

The VeClose trial is a multicenter, prospective randomized controlled trial executed at 10 participating centers in the United States under an investigational device exemption approved by the FDA. The subjects were enrolled between March and September 2013. All the study centers obtained approvals from a central Institutional Review Board before patient enrollment and each subject provided informed consent after his/her eligibility was confirmed. A detailed description of the study design, eligibility criteria, and outcome measures was reported previously in the publication of the three-month results.^11^ Twelve- and 24-month results have also been reported.^5,12^ This trial included adult patients with symptomatic venous reflux and varicosities who had clinical–etiology–anatomy–pathophysiology (CEAP) classifications of C2–C4b and GSV incompetence with a reflux time of ≥0.5 s assessed in the standing position using duplex ultrasound. Patients were excluded if they were asymptomatic, had clinically significant reflux of the SSV or anterior accessory GSV, previous treatment of venous disease in target limb, symptomatic peripheral arterial disease, a history of deep venous thrombosis, pulmonary embolism, or aneurysm of the target GSV with a diameter of >12 mm. All the participants underwent baseline examination including physical examination, completion of CEAP classification and Venous Clinical Severity Score (VCSS) assessments,^14^ and duplex ultrasound examination of both legs. They also completed the Aberdeen Varicose Vein Questionnaire (AVVQ) and EQ-5D QoL survey.^15,16^ At the baseline visit, each investigator was asked to predict, based on their own extensive thermal ablation experience, whether adjunctive treatment would be necessary and if so the extent of such treatment.

### Randomization

Two hundred forty-two subjects were enrolled in this trial, of whom 222 patients were randomized (1:1) to either CAC (VenaSeal; *n* = 108) or RFA (ClosureFast; *n* = 114) (both now manufactured by Medtronic, Minneapolis, MN, USA). Randomization was stratified by study center and random block sizes of four or six were used; assignments were obtained through automated telephone service connected to a password-protected randomization table. The first two cases at each site (non-randomized) were used as roll-in cases and were treated with CAC to train and ensure familiarity with the procedure.^11^

### Devices and procedures

Endovenous treatment of the GSV with the CAC device was performed as previously described,^11^ following the instructions for use. Manual compression over the vein during the polymerization phase is used to eliminate as much blood as possible from the vein to achieve complete adhesion of the cyanoacrylate to the vein wall.

Subjects in the RFA group were treated using the ClosureFast™ system according to the instructions for use. Post-procedure compression stockings were used for three days continuously and an additional four days during waking hours in both groups. No adjunctive treatments were allowed during the first three months in either the ipsilateral or contralateral leg. The decision to perform adjunctive procedures, and which procedures would be utilized was made in consultation between physician and patient at each follow-up visit. Adjunctive treatment was directed to residual varicosities or the untreated portion of the GSV.

### Post-procedural follow-up

Following the treatment, subjects visited the investigators’ clinic at day 3 and at 1, 3, 6, 12, 24, and 36 months for clinical assessments. At the day 3 visit, VCSS, a duplex ultrasound exam, an evaluation for ecchymosis and post-procedural pain scoring were performed. During the follow-up visits, the investigators completed VCSS at day 3, CEAP assessments from three months onwards and AVVQ and EuroQol 5 Dimension (EQ-5D) QoL surveys. The EQ-5D (health thermometer) was assessed by the visual analogue scale (VAS) technique wherein respondents were asked to indicate where they should position their health state on a vertical thermometer-like scale ranging from best imaginable to worst imaginable health. In addition, duplex ultrasound exam of the treated vein was performed by a registered vascular technologist and interpreted by each investigator and, for the initial three months, by the core lab. The interpretations of the core lab and the investigators were in 100% agreement for closure of the target veins. Patients completed a brief questionnaire about treatment satisfaction, which included responses like “very dissatisfied”, “somewhat dissatisfied”, “somewhat satisfied”, and “very satisfied” with the treatment provided. The occurrence of adverse events (AEs) was also assessed at each follow-up visit. Investigators rated the event severity and the relationship of the AEs to the device/procedure. Safety was reviewed by an independent Data Safety Monitoring Board up to the 12-month visit. The incidence of the following types of AEs – serious, non-serious, and unanticipated events, deep vein thromboses in either leg, pulmonary embolism, and AEs occurring in the treated limb were assessed from the 12-month follow-up visit until the completion of the 36-month follow-up visit.

### Statistical analysis

The primary outcomes are the complete closure of target GSV at 36 months and time to recanalization through 36 months. The complete closure of the target GSV at the 36-month visit was defined as duplex ultrasound examination (including color flow, compression, and pulsed Doppler) showing closure along the entire treated target vein segment with no discrete segments of patency exceeding 5 cm in length.^11^ Proportion of subjects with complete closure of the target vein in the two groups at each visit through month 36 were compared using a non-inferiority approach with *P-*values calculated based on the Farrington–Manning method.^17^ The trial’s non-inferiority margin for the primary endpoint at month 3 was 10%. The same non-inferiority margin was used for the other time point assessment. Recanalization was defined as patency along the treated segment exceeding 5 cm in length.^11^ Time to recanalization was calculated as the number of days from treatment to first instance of recanalization. The freedom from recanalization was estimated using Kaplan–Meier method and compared using log-rank test. The secondary outcomes were the changes in VCSS, AVVQ score, and EQ-5D score from baseline to month 36 and the occurrence of AEs and serious adverse events (SAEs). Changes from baseline in VCSS, AVVQ score, and EQ-5D score were compared between the two treatment groups using repeated measures analysis of variance. Wilcoxon test was used to compare patient satisfaction between the two treatment groups. Each investigator was asked to predict the need for the type and extent of adjunctive treatment before any treatment was instituted (predictive), and to track adjunctive treatment following endovenous ablation (actual). The predictive and actual need for adjunctive treatment at baseline and at follow-up (6, 12, 24, and 36 months) were compared between the two groups. Fisher’s exact test was used to compare the AE rates. All the analyses were performed using SAS 9.4 (SAS Institute Inc., Cary, North Carolina, USA). *P-*values of <0.05 were considered as statistically significant.

## Results

### Baseline characteristics of the study population

The randomized portion of this trial included 222 eligible subjects treated between March and September 2013,^11^ of whom 108 were assigned to CAC and 114 to RFA. There were no statistically significant differences in the baseline characteristics between the two treatment groups (Table 1). The majority of participants (87%) were in the CEAP classifications C2 or C3 at baseline.

**Table 1.** Baseline characteristics of the VeClose trial subjects.

Characteristics	CAC (*N* = 108)	RFA (*N* = 114)	*P-*value
Age, mean (range)	49.0 (26.6–70.6)	50.5 (25.6–70.1)	0.34
Body mass index, mean (range)	27.0 (17.4–44.5)	27.0 (17.0–46.7)	0.95
Primary symptoms, *n* (%)			0.65
Pain	33 (31)	24 (21)	
Aching	32 (30)	39 (34)	
Itching	2 (2)	5 (4)	
Burning	5 (5)	3 (3)	
Heaviness	14 (13)	16 (14)	
Swelling	17 (16)	18 (16)	
Others	4 (4)	7 (6)	
GSV diameter, mean (*n*), mm			
Mid GSV	4.9 (0–9)	5.1 (2.4–11)	0.22
Proximal GSV	6.3 (3–12)	6.6 (2.8–12)	0.30
CEAP classification, *n* (%)			0.96
C2	61 (57)	64 (56)	
C3	32 (30)	36 (32)	
C4a	13 (12)	12 (11)	
C4b	2 (2)	2 (2)	
VCSS, mean (SD)	5.5 (2.6)	5.6 (2.6)	0.99
AVVQ, mean (SD)	18.9 (9.0)	19.4 (9.9)	0.72
EQ-5D health thermometer, mean (SD)	83.5 (16.3)	84.9 (12.3)	0.48

AVVQ: Aberdeen Varicose Vein Questionnaire; CAC: cyanoacrylate closure; CEAP: clinical, etiologic, anatomic, pathophysiologic; EQ-5D health thermometer as assessed by visual analogue score (VAS); GSV: great saphenous vein; RFA: radiofrequency ablation; SD: standard deviation; VCSS: Venous Clinical Severity Score.

### Study outcomes

#### Patient follow-up

At 36 months, 72 out of 108 subjects in the CAC group (67%) and 74 out of 114 subjects in the RFA group were evaluated. The lack of data for approximately one-third of patients in each group was due to patient dropout or the data could not be collected in the time period dictated by the study period.

#### GSV closure rate

The GSV closure rate at month 36, as judged by the investigator, was slightly higher with CAC (94.4%, 68/72) than that with RFA (91.9%, 68/74), although not statistically significant (Table 2). The closure rates at months 3, 6, 12, and 24 were reported to be 99%, 99%, 96.8%, and 95.3% for the CAC group and 95.4%, 96.2%, 95.9%, and 94.0% for the RFA group, respectively, with non-inferiority shown at each time period (Table 2).

**Table 2.** GSV closure rate at different time points as judged by the investigator.

Timepoints	CAC	RFA	*P-*value^1a^	*P-*value^2b^
Day 3	100% (108/108)	99.1% (113/114)	0.0001	1.00
Month 1	100% (105/105)	87.3% (96/110)	<0.0001	<0.0001
Month 3	99% (103/104)	95.4% (103/108)^c^	<0.0001	0.22
Month 6	99% (100/101)	96.2% (101/105)	0.0001	0.37
Month 12	96.8% (92/95)	95.9% (93/97)	0.0015	1.00
Month 24	95.3% (82/86)	94.0% (79/84)	0.0034	0.75
**Month 36**	**94.4% (68/72)**	**91.9% (68/74)**	**0.0050**	**0.75**

CAC: cyanoacrylate closure; GSV: great saphenous vein; RFA: radiofrequency ablation.

a One-sided *P-*value for non-inferiority comparing CAC and RFA with 10% margin.

b Two-sided *P-*value comparing CAC and RFA using Fisher’s exact test.

c Minor decrease from the three-month manuscript^11^ due to data corrections made at the investigational site.Note: Bold indicates results at 36 months, the subject of this submission.

### Survival free from recanalization

**Figure 1.**
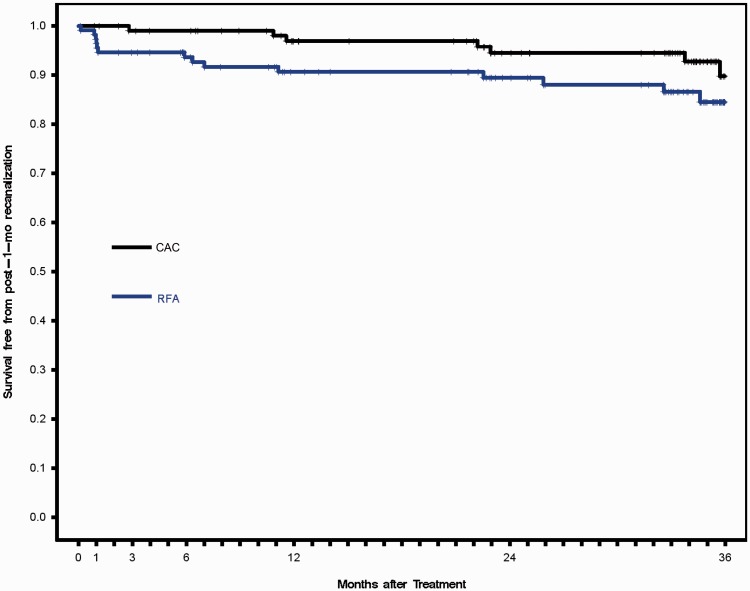
Survival from recanalization of the target vein for the CAC and RFA treatment groups. Black line = CAC; blue line = RFA.

When compared with the CAC group, the RFA group showed a numerically lower rate of freedom from recanalization throughout the study period, i.e. the probability of recanalization was higher, even though the difference was not statistically significant (log-rank, *P* = 0.1006) (Figure 1).

### Symptom scores and QoL

**Figure 2.**
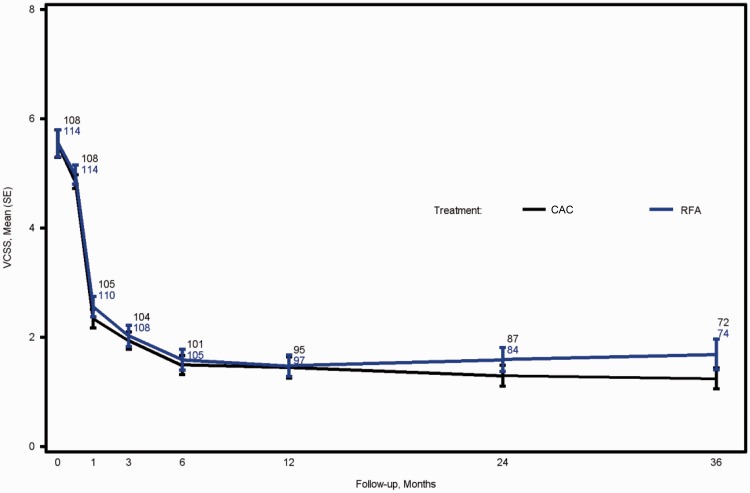
Change in VCSS in the treatment groups over 36 months. Data points represent the number of patients with available data.

**Figure 3.**
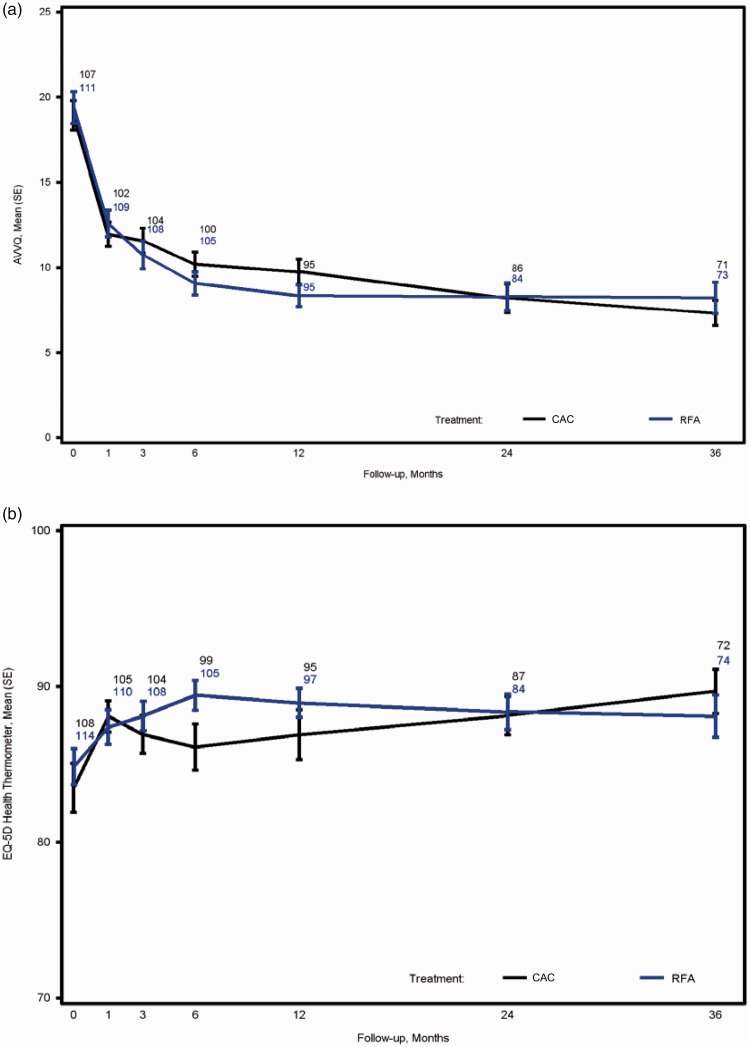
Change in AVVQ and EQ-5D health thermometer in the treatment groups over 36 months. The change in AVVQ (a) and EQ-5D health thermometer as assessed by VAS (b) are shown by time for the CAC and RFA treatment groups. Black line = CAC; blue line = RFA. SE: standard error. Data points represent the number of patients with available data.

VCSS of the two treatment groups were comparable at baseline and declined over time for all subjects with no significant difference between the groups. Maximum improvement in the VCSS was observed at month 6 and persisted to month 36 in both groups (Figure 2). Similarly, there was no statistical difference between CAC and RFA treatment groups in both AVVQ (*P* = 0.45) (Figure 3(a)) and EQ-5D health thermometer as assessed by VAS (*P* = 0.42) (Figure 3(b)) which remained improved throughout the 36-month period.

### Adjunctive treatments

Sclerotherapy and ambulatory phlebectomy or other procedures were used if needed as adjunctive treatments after the 3-month visit and at the 6-, 12-, 24-, and 36-month follow-up visits to eliminate persistently incompetent untreated GSV segments and residual varicosities. After the three-month visit but before the six-month visit, two patients (1.9%) in the RFA group required additional sclerotherapy compared to none in the CAC group (*P* = 0.498). In the CAC and RFA groups, at the six-month visit, sclerotherapy was applied in 66.3% (69/104) and 63.9% (69/108) of the subjects, respectively (*P* = 0.774) while phlebectomy was applied in 17.3% (18/104) and 19.4% (21/108) of the subjects, respectively (*P* = 0.726). The rates of additional adjunctive sclerotherapy and phlebectomy treatments decreased considerably from 12 to 36 months following both CAC and RFA treatments with phlebectomy being carried out in 4.2% (3/72) of the subjects in the CAC group but none (0/73) in the RFA group (*P* = 0.120) (data not shown as table).

In the CAC and RFA groups, the predicted number of adjunctive treatments at baseline was compared with the actual number of adjunctive procedures done in follow-up visits at 6, 12, 24, and 36 months in the intent to treat population (Table 3). The data analysis revealed that at six months, the actual number of adjunctive sclerotherapy treatments carried out was significantly lower than that predicted at baseline in both the CAC and RFA groups (*P* = 0.02 for both). At 12-, 24-, and 36-month follow-up, the actual number of adjunctive sclerotherapy treatments in the CAC group was significantly lower than that predicted at baseline (*P* = 0.03, *P* = 0.04, and *P* = 0.04, respectively) but the difference was not statistically significant in the RFA group. The actual number of adjunctive phlebectomy treatments at all time periods was significantly lower than what was initially predicted at baseline (*P* < 0.0001) in both CAC and RFA groups. Overall, there were no significant differences between the groups in the requirement for adjunctive therapies at follow-up.

**Table 3.** Predicted and actual adjunctive procedures (sclerotherapy and phlebectomy) by visit in the intent to treat population.

Treatment	CAC (*N*=108)	RFA (*N*=114)
Sclerotherapy		
Estimated number at baseline	7.78±18.20	6.87±16.08
Actual number (by six months)	3.57±3.75	3.58±4.22
* P*-value^a^	0.02	0.02
Actual number (by 12 months)	4.06±3.95	4.50 ±6.05
* P*-value^a^	0.03	0.07
Actual number (by 24 months)	4.34±4.64	4.61±6.06
* P-*value^a^	0.04	0.09
Actual number (by 36 months)	4.37±4.67	4.65±6.12
* P-*value^a^	0.04	0.09
Phlebectomy		
Estimated number at baseline	4.58±5.58	4.11±5.15
Actual number (by six months)	1.45±4.20	1.56±3.98
* P-*value^a^	<0.0001	<0.0001
Actual number (by 12 months)	1.45±4.20	1.65±4.00
* P-*value^a^	<0.0001	<0.0001
Actual number (by 24 months)	1.45±4.20	1.65±4.00
* P-*value^a^	<0.0001	<0.0001
Actual number (by 36 months)	1.86±5.06	1.65±4.00
* P*-value^a^	<0.0001	<0.0001

Data represented as mean±SD. CAC: cyanoacrylate closure; RFA: radiofrequency ablation.

a Two-sided *P-*value comparing the baseline estimated number and actual number throughout the trial was calculated using paired t-test.

### Patient satisfaction

At 36 months, patients belonging to both the treatment groups were “somewhat satisfied” to a similar extent, whereas 84.7% (61/72) of the CAC group and 78.4% (58/74) of the RFA group were “very satisfied” with the treatment. The *P*-value for the difference in satisfaction is 0.30 (data not shown as table).

### Adverse events

A total of seven non-serious AEs in the target/ipsilateral limb (five events from the CAC group and two from the RFA group) occurred in seven subjects between months 24 and 36 (Table 4). Of the total seven AEs reported, one subject reported a scar (possibly attributed to slight glue retention at exit site) which was related to the CAC study device and one subject reported late onset of phlebitis, which was possibly related to the CAC procedure. In addition to the AEs, a total of six SAEs (four events from the CAC and two from the RFA group) occurred in four subjects between months 24 and 36 (Table 5). No deep vein thrombi, pulmonary emboli, or unanticipated AEs occurred during this period.

**Table 4.** Non-serious AEs in the target limb by group and time period in the VeClose trial (24–36 months).

Treatment	AE description	Related to device	Related to procedure	Days to AE
CAC	1.5-cm cystic mass in index leg	NR	NR	735
CAC	Acute ankle pain	NA	NA	788
CAC	Late onset of phlebitis	NR	PR	976
CAC	Scar	DR	DR	1175
CAC	Left leg calf pain	NR	Unknown	1241^a^
RFA	Non-treatment zone phlebitis	NR	NR	1062
RFA	Superficial phlebitis right leg	NR	NR	1062

AE: adverse event; CAC: cyanoacrylate closure; DR: definitely related; NA: not applicable; NR: not related; PR: probably related; RFA: radiofrequency ablation.

a Included in the current report due to the late completion of the 36-month visit.

**Table 5.** SAEs by group and time period in the VeClose trial (24–36 months).

Treatment	AE description	Related to device	Related to procedure	Days to AE
CAC	Liver cancer	NR	NR	756
CAC	Breast invasive lobular Carcinoma	NR	NR	863
CAC	Cervical pain	NR	NR	915
CAC	Suicide attempt	NR	NR	1030
RFA	Knee arthroplasty in the contralateral leg^a^	NR	NR	734
RFA	Knee arthroplasty in the ipsilateral/index leg^a^	NR	NR	816

SAE: serious adverse event; CAC: cyanoacrylate closure; NR: not related; RFA: radiofrequency ablation.

^a^Arthroplasty in the same patient at different time periods.

## Discussion

The promising technique of CAC embolization of incompetent truncal veins has been demonstrated to be safe and effective when compared with RFA treatment in the previous reports from the VeClose trial at 3, 12, and 24 months.^5,11,12^ The results of this 36-month follow-up trial are consistent with the earlier clinical reports, further confirming the effectiveness of CAC compared with the RFA treatment. The 36-month occlusion rate (94.4%) of the target GSV with CAC in this trial is similar to the rate reported in a three-year follow-up, first-human-use CAC study (94.7%).^9^ The closure rate of CAC found in this trial is also comparable to that of previous studies that used other techniques for GSV closure. In an RFA trial, the GSV closure rate at 36 months was 92.6% by Kaplan–Meier analysis^18^ and in a randomized clinical trial that compared four treatment groups, the closure rates at three years after treatment were 93% for RFA, 93.5% for stripping, 93.2% for EVLT, and 73.6% for foam sclerotherapy.^19^ The five-year report of the same trial showed closure rates of 93.2% for RFA, 93.7% for stripping, 93.2% for EVLT, and 68.4% for sclerotherapy.^20^

Although the GSV closure rates were comparable between the two treatment groups in the VeClose trial, the use of CAC was beneficial compared with RFA because neither TA nor thermal energy is required.^11^ Compression stockings were used in both treatment groups in this trial to avoid bias, though they are not routinely used following CAC. Other studies^4,7,10^ did not require post-procedure compression in subjects treated with CAC and had similar findings.

Endovenous methods of treatment for saphenous vein reflux commonly initially result in thrombosis of the target vein. However, since thrombosis is frequently associated with varying degrees of recanalization, there must be sufficient chemically or thermally induced vein wall destruction, in addition to the thrombosis, to achieve fibrosis of the vein and permanent occlusion.

**Figure 4.**
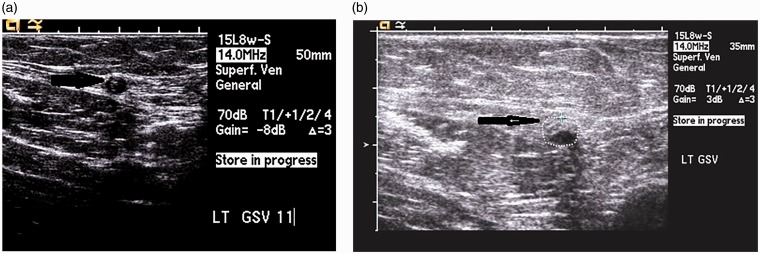
(a) Transverse view of great saphenous vein (GSV) at one-month post-treatment (black arrow points to occluded vein). (b) Transverse view of great saphenous vein (GSV) at one-year post-treatment (black arrow points to occluded vein). Images reproduced with permission of Morrison Vein Institute.

On the contrary, CAC does not produce significant thrombosis because the vein walls are immediately coapted to the adhesive by the application of external compression resulting in an inflammatory and eventual fibrotic reaction rather than a thrombotic one.^21^ Ultrasound images of the target vein at one month and one year following CAC demonstrate essentially no reduction in size (Figure 4(a) and (b)), whereas ultrasound images of chemically or thermally treated veins demonstrate complete disappearance of the vein. The fact that minimal visible reduction in the size of the CAC-treated vein is seen on ultrasound imaging further supports the notion that thrombosis is not a component of CAC vein destruction.

Overall, at all the follow-up visits, less frequent target vein recanalization (defined as patency along the treated segment exceeding 5 cm in length) was observed in CAC (7) compared with RFA (14). Of the seven subjects with recanalization in the CAC group, one vein each opened at the end of 3 and 12 months, two veins between 12 and 24 months, and three veins between 24 and 36 months. Of the 14 subjects with recanalization in the RFA group, six veins opened at the end of 3 months, four veins at 12 months, one vein between 12 and 24 months, and three veins between 24 and 36 months. However, it is important to note that patient-reported and QoL outcomes did not change despite these recanalizations.

As expected, anatomic success in closure of incompetent GSVs was related to clinical improvements. Significant improvements in VCSS were observed from baseline through 36 months in both the CAC and RFA groups, which were similar to results seen in prior study.^18,19^ Likewise, significant and sustained improvements in AVVQ and EQ-5D (VAS) from baseline through 36 months were observed in both treatment groups. The persistent clinical benefit was further indicated by patient satisfaction in both treatment groups. Patient satisfaction was higher in the CAC group than that in the RFA group at three years, though not significant, with the majority of patients from both treatment groups stating they would have the same procedure again if given the opportunity.

Phlebectomy and sclerotherapy are adjunctive treatments routinely used in clinical practice.^22^ Both were used in this trial; however, both ipsilateral and contralateral post-operative adjunctive treatments were not performed within the first three months after the initial CAC or RFA treatment to minimize potential confounding of the outcome measurements.^11^ The number of actual adjunctive treatments carried out at 12, 24, and 36 months in the CAC group was significantly lower than what was predicted at baseline, whereas in the RFA group it was marginally lower. However, there were no significant differences between groups in the overall requirement for adjunctive therapies at follow-up. Because of the high viscosity of VenaSeal™ adhesive, migration into tributaries was not seen, and thus the number of adjunctive procedures performed in both groups was roughly equal. This further confirms CAC was comparable to RFA in clinical improvement when used as a stand-alone procedure.

The rate of AE occurrence was similar among the two treatment groups. During months 24–36, there were only two AEs reported in the CAC group and no device-related or procedure-related AEs in the RFA group. One case of late onset phlebitis was CAC procedure-related while the case of scar was directly related to the both CAC procedure/device. Although not statistically significant, phlebitis or a general inflammatory response along the treated vein was more common in the CAC group.^5^ This was treated solely with non-steroidal anti-inflammatory drugs in both groups and was self-limiting. No pulmonary emboli or deep venous thrombi occurred in either group. SAEs such as liver cancer, right breast invasive lobular carcinoma, cervical pain, and suicide attempt were reported in the CAC group, whereas knee arthroplasty in the ipsilateral (index limb) and contralateral legs was observed in the RFA group. However, none of these SAEs were related to the device or the procedure.

The strengths of this trial include its randomized controlled design in addition to diligent measurement of clinical outcomes and symptom scores. All data were reviewed by external monitors and 100% source verified, yielding high-quality results. The correlation between the investigator and the core laboratory was 100% at three months. In addition, the multicenter, multi-operator performance of the trial increases the validity by removing the bias of a single-center/operator trial and describing the combined experience of multiple centers. Finally, adjunctive treatments were withheld until after the three-month visit to prevent confounding variables in the primary occlusion analysis. The follow-up will continue to 60 months post-procedure to assess long-term results.

The current trial has a few limitations. Although blinding is potentially advantageous, it was not entirely feasible, as RFA requires TA administration. The trial is also limited by relatively high number of patient with missing data from each treatment group. And finally, potential advantages of CAC over RFA were purposefully not addressed in this study, as the present report is only intended to demonstrate 36-month parity of CAC with RFA. Some advantages might include: immediate return to normal activity without waiting for the local anesthetic effect to resolve; lack of compression hose in warm or humid climates; physical limitations to donning and doffing of compression hose in older or overweight individuals leading to non-compliance; and technical challenges in thermal ablation techniques such as staff time and equipment to mix tumescent solution, shortages of mixing fluids (e.g. saline), malfunctioning generators or catheters. Cost analysis comparisons of CAC and RFA were not included in the design of the study, but could be forthcoming in future studies in addition to analyses of the potential advantages of CAC described above.

## Conclusions

The present trial reports similar GSV closure rates with both CAC and RFA at 36 months, further confirming the durability and non-inferiority of CAC compared to RFA. In addition, VCSS and QoL significantly improved from baseline to six months and were maintained at 36 months in both treatment groups. In both the groups, SAEs were not related to either the device or the procedure, and patient satisfaction was high. Since CAC does not require the use of TA or thermal energy, demonstration of this longer term safety and effectiveness further highlights the benefits of this non-thermal approach for patients with incompetent saphenous veins. Follow-up of the patient cohorts post-procedure will continue up to 60 months.
